# The gut–brain axis as a pivotal regulator in autoimmune pathogenesis

**DOI:** 10.1097/MS9.0000000000003965

**Published:** 2025-09-23

**Authors:** Rahmah Zafar Masood, Muneeba Ali, Syeda Faiqa Manzoor, Emad Uddin Sajid, Christian Tague, Hermann Yokolo, Aymar Akilimali

**Affiliations:** a CMH Lahore Medical College, Lahore, Pakistan; b Department of Medicine, Karachi Medical and Dental College, Karachi, Pakistan; c Department of Medicine, Dow University of Health Sciences, Karachi, Pakistan; d Department of Research, Medical Research Circle (MedReC), Goma, DR Congo

**Keywords:** autoimmune pathogenesis, gut–brain axis, pivotal regulator

*Dear Editor*,

The rapidly evolving understanding of the gut–brain axis (GBA) has revealed its fundamental role in the pathophysiology of autoimmune disorders. As a complex bidirectional communication network integrating neural, endocrine, and immunological pathways between the gastrointestinal tract and central nervous system, the GBA provides new insights into disease mechanisms and possible therapeutic interventions for conditions including multiple sclerosis (MS), rheumatoid arthritis (RA), and systemic lupus erythematosus (SLE)^[1]^. This article complies with the TITAN 2025 guidelines – repressing the reporting and use of AI^[2]^.

There is growing evidence that autoimmune pathogenesis is significantly impacted by gut microbiota dysbiosis. Detailed metagenomic investigations have identified specific microbial patterns associated with several autoimmune conditions. Specific alterations in the gut microbiota, including decreased *Faecalibacterium prausnitzii* and increased *Akkermansia muciniphila* in MS, as well as overrepresentation of *Prevotella copri* in RA, have been associated with autoimmune pathogenesis. These bacteria can trigger an immune response against the host via several mechanisms. Molecular mimicry involves bacterial antigens resembling human proteins, thereby activating autoreactive T cells. Bystander activation allows nonspecific stimulation of T cells in an inflammatory context, amplifying autoimmunity. Finally, epitope spreading progressively broadens the immune response to new self-antigens, promoting disease chronicity. These mechanisms demonstrate the key role of the intestinal microbiota as a regulator of immune tolerance and contribute to the initiation and progression of autoimmune diseases^[1,3]^. These microbial changes seem to trigger autoimmunity via multiple mechanisms, including molecular mimicry, epitope spreading, and bystander activation of autoreactive lymphocytes.

Another significant factor in GBA-mediated autoimmunity is the integrity of the intestinal epithelial barrier. Accumulated intestinal permeability allows lipopolysaccharide (LPS), a bacterial endotoxin, to activate Toll-like receptor 4 (TLR4) on immune cells. This activation triggers the nuclear factor kappa B (NF-κB) signaling pathway, leading to the production of pro-inflammatory cytokines such as interleukin-6 (IL-6), interleukin-17 (IL-17), and interleukin-23 (IL-23). These cytokines underline the differentiation of T helper 17 (Th17) cells and inhibit the development of regulatory T (Treg) cells, thus disrupting the immune balance. Excessive NF-κB activation, particularly via the RelA subunit, directly regulates the expression of RAR-related orphan receptor gamma t (RORγt), a key Th17 transcription factor. This molecular cascade contributes to chronic inflammation and the progression of autoimmune diseases^[4,5]^. This phenomenon has been well-documented in SLE, where elevated serum LPS levels predict the development of flares and are correlated with the disease severity^[6]^.

Neuroimmune interactions mediated by the vagus nerve constitute a third major component of GBA involvement in autoimmunity. The anti-inflammatory cholinergic pathway, also known as the anti-inflammatory vagal pathway, is a neuroimmune mechanism where stimulation of the vagus nerve releases acetylcholine. This molecule binds to the α7 nicotinic acetylcholine receptor (α7nAChR) on macrophages, thereby inhibiting the production of pro-inflammatory cytokines such as tumor necrosis factor α (TNF-α) and IL-6. This process is mediated by JAK2-STAT3 pathway signaling and NF-κB pathway activation. Vagal stimulation, for example via implantable devices, has shown therapeutic potential in experimental models of chronic inflammatory diseases, suggesting its interest in the treatment of autoimmune pathologies. These findings pave the way for new therapeutic strategies modulating nerve communication to control systemic inflammation^[7,8]^. On the other hand, worse outcomes in several autoimmune diseases are predicted by reduced vagal tone, as indicated by heart rate variability. This neuroimmunological axis may explain the well-documented but poorly understood connection between psychological stress and autoimmune exacerbations^[9]^. Chronic stress activates the hypothalamic–pituitary–adrenal axis, leading to the release of glucocorticoids (cortisol) and catecholamines (adrenaline and noradrenaline). These hormones modulate immune cell function by regulating the production of pro-inflammatory cytokines, thereby disrupting immune balance. They also affect the composition of the gut microbiota, contributing to dysbiosis that impairs the intestinal barrier and promotes systemic inflammation. This interaction between stress, the immune system, and the gut microbiota creates a vicious cycle, exacerbating chronic inflammatory diseases and metabolic disorders such as type 2 diabetes^[10]^ (Table 1).

**Table 1:** Mechanisms linking GBA dysregulation to autoimmune disease

Mechanism	Key players/Molecules	Process/Effect	Examples/Notes
Microbiota dysbiosis	*F. prausnitzii, A. muciniphila, P. copri*	Molecular mimicry: bacterial antigens resemble host proteins → activation of autoreactive T cells; Bystander activation: nonspecific T cell stimulation in inflammation; Epitope spreading: immune response broadens to new self-antigens	MS: ↓*F. prausnitzii*, ↑*A. muciniphila*; RA: ↑*P. copri*
Leaky gut/barrier dysfunction	LPS, TLR4, NF-κB, IL-6, IL-17, IL-23, Th17, Treg, RORγt	Increased intestinal permeability allows LPS translocation → TLR4 activation → NF-κB signaling → pro-inflammatory cytokine release → Th17 differentiation, Treg inhibition → chronic inflammation	Documented in SLE: elevated serum LPS correlates with flares and disease severity
Vagal anti-inflammatory pathway	Acetylcholine, α7nAChR, JAK2-STAT3, NF-κB, TNF-α, IL-6	Vagal stimulation releases acetylcholine → binds α7nAChR on macrophages → inhibits pro-inflammatory cytokine production → reduces systemic inflammation	Reduced vagal tone predicts worse outcomes in autoimmune diseases; therapeutic potential via implantable devices
Stress circuit/Neuroendocrine influence	Cortisol, Catecholamines (adrenaline, noradrenaline)	Chronic stress activates HPA axis → stress hormones modulate immune cells → increase pro-inflammatory cytokines, disrupt immune balance → alter gut microbiota → worsen barrier function → vicious cycle	Exacerbates chronic inflammatory and metabolic diseases (e.g., type 2 diabetes)

These mechanistic insights have spawned numerous therapeutic investigations. Microbiota-targeted interventions, including specific probiotic formulations (particularly those containing *Lactobacillus* and *Bifidobacterium* strains), prebiotic fibers, and fecal microbiota transplantation, have shown promising results in early clinical trials. Dietary modifications emphasizing anti-inflammatory nutrients and polyphenols appear to synergize with microbial therapies. Pharmacological approaches targeting intestinal barrier function, such as zonulin inhibitors, are under active development. Perhaps most intriguingly, noninvasive vagus nerve stimulation devices are now being evaluated in multicenter trials for autoimmune indications.

The clinical implications of GBA research extend beyond therapeutics to include diagnostic and preventive strategies. Microbial signatures may enable earlier disease detection and more accurate prognostic stratification. In high-risk populations, microbiome modulation could potentially delay or prevent the onset of autoimmune disease. These applications are particularly relevant given the dramatic increase in the prevalence of autoimmune disease over recent decades, a trend that parallels Western lifestyle changes impacting gut microbiota composition.

Despite tremendous advancements, there are still critical knowledge gaps. Clarification is needed about the relative contributions of specific microbial taxa versus functional changes at the community level. Longitudinal studies are required to establish causal relationships between GBA alterations and disease progression. The development of standardized, reproducible interventions represents another key challenge.

In conclusion, the gut–brain axis plays a key role in autoimmune diseases, integrating environmental, microbial, and neuroimmune factors. This fundamental change in our understanding of autoimmunity creates intriguing opportunities for therapeutic innovation and research. We urge increased investment in GBA-focused studies and accelerated translation of these findings into clinical practice. One of contemporary medicine’s most promising areas is the possibility for GBA-targeted approaches to revolutionize the management of autoimmune diseases.

## Data Availability

Not applicable.
